# Pathogen-based precision medicine for drug-resistant tuberculosis

**DOI:** 10.1371/journal.ppat.1007297

**Published:** 2018-10-18

**Authors:** Matthias I. Gröschel, Timothy M. Walker, Tjip S. van der Werf, Christoph Lange, Stefan Niemann, Matthias Merker

**Affiliations:** 1 Molecular and Experimental Mycobacteriology, Research Center Borstel, Borstel, Germany; 2 Department of Pulmonary Diseases & Tuberculosis, University Medical Center Groningen, University of Groningen, Groningen, The Netherlands; 3 Nuffield Department of Medicine, University of Oxford, John Radcliffe Hospital, Oxford, United Kingdom; 4 Clinical Infectious Diseases, Research Center Borstel, Borstel, Germany; 5 International Health / Infectious Diseases, University of Lübeck, Lübeck, Germany; 6 Department of Medicine, Karolinska Institute, Stockholm, Sweden; 7 German Center for Infection Research (DZIF) Tuberculosis Unit, Borstel, Germany; Tufts Univ School of Medicine, UNITED STATES

## Introduction

**Fig 1:**
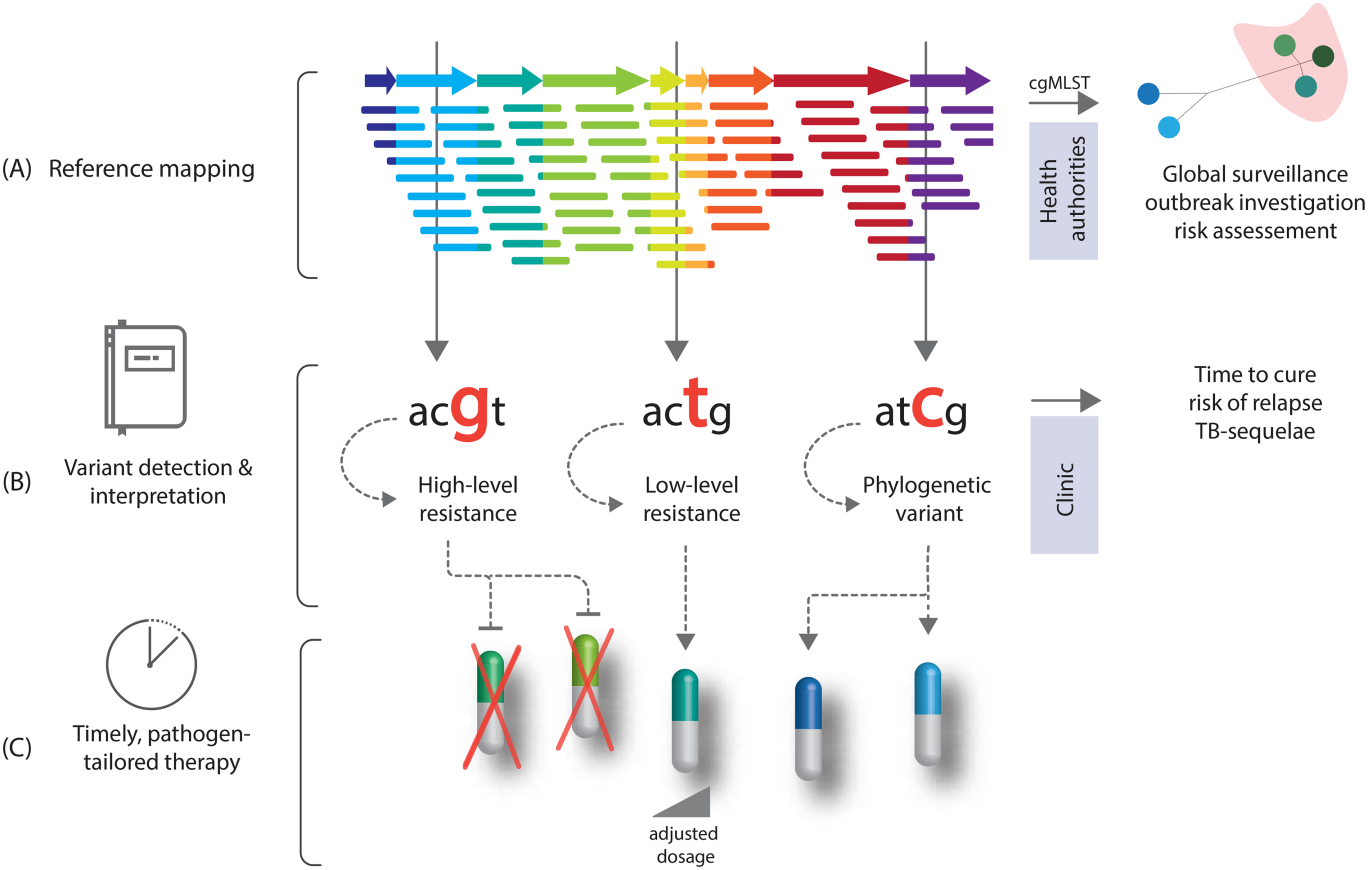
Principle of pathogen-tailored individualized treatment design. (A) Mutations are obtained from a whole-genome sequencing reference mapping approach that can be also transferred into a cgMLST for molecular outbreak surveillance. (B) Individual mutations are further interpreted towards their biological phenotype employing a validated consensus mutation catalogue. (C) When canonical and/or high-level resistance-conferring mutations are present, this drug should not be used. However, mutations associated with a moderate or intermediate resistance level may allow the use of drugs at increased doses. Moreover, mutations can be used to predict different treatment outcomes. Thus, by also considering phylogenetic benign mutations that do not confer resistance, a comprehensive molecular drug susceptibility profile could be inferred for a pathogen-tailored individualized treatment regimen in the future. cgMLST,core genome multilocus sequencing type; TB, tuberculosis.

The implementation of next generation sequencing techniques, such as whole-genome sequencing (WGS), in tuberculosis (TB) research has enabled timely, cost-effective, and comprehensive insights into the genetic repertoire of the human pathogens of the *Mycobacterium tuberculosis* complex (MTBC). WGS data allow for detailed epidemiological analysis based on genomic distance of the MTBC strains under investigation, e.g., for tracing outbreaks; it can accelerate diagnostics by predicting drug resistance from a mutation catalogue (Fig 1). Indeed, specific mutations even permit predictions on the possible clinical treatment course and outcome [1–4].

These examples of how genomic data inform treatment choice illustrate the concept of precision medicine in infectious diseases, in which prevention and treatment strategies take information from systems biology and individual variability into account [5]. With the increasing volume of biological and genomic sequence data of pathogens, precision medicine in infectious diseases has gained momentum in recent years.

In this Pearl, we briefly portray how genome sequencing has transformed and accelerated delivery of tailored treatment to patients with multidrug-resistant (MDR)-TB (defined by in vitro drug resistance against rifampin and isoniazid) and extensively drug-resistant (XDR)-TB (defined by MDR-TB plus in vitro drug resistance against a fluoroquinolone and a second-line injectable drug—amikacin, capreomycin, or kanamycin). We describe its potential to infer drug resistance profiles and forecast treatment outcomes. Although implementation of personalized TB therapy may seem difficult under programmatic conditions, genome-based resistance and outcome prediction are likely to become feasible for this purpose in the near future.

## MDR-TB as a model example of precision medicine in infectious diseases

The emergence of drug-resistant MTBC strains is a major public health challenge. WHO reports 72% and 65% of MDR-TB among previously treated TB cases in Belarus and the Russian Federation, respectively [6]. The burdening treatment of MDR-TB/XDR-TB takes much longer, yielding successful outcome in around 60% in MDR-TB and 35% in XDR-TB patients [7,8]. Treatment regimens are empirical from the start—later to be modified as per phenotypic drug susceptibility test (pDST) results. This process can take several weeks to months due to slow mycobacterial growth. While classical point-of-care molecular tests, as discussed later, provide rapid information on selected mutations on few drugs, the treatment already started may contain ineffective and potentially toxic drugs until pDST results become available [9]. By leveraging the genomic information of the infecting bacilli, individualized and precise treatment regimens can be tailored to the resistance profile of the infecting microorganism of a particular patient, instead of using a standardized drug combination. MDR-TB thus serves as a model example of how the individual variability of the infecting pathogen is taken into account to deliver faster, precise, and more effective treatment to patients.

## Classical molecular test to inform MDR-TB and XDR-TB therapy

The fundamental concept underlying any molecular test is to predict a biological phenotype based on the genetic mutation or variant detected [10]. These drug resistance-conferring variants in the genome present reliable biomarkers to predict drug resistance and to design effective treatment regimens without awaiting culture results. Molecular tests to quickly identify genotypic drug resistance have been successfully implemented in daily practice [11]. The first molecular assays widely introduced were the line probe assays such as the Genotype MTBDRplus and MTBDRsl. These qualitative in vitro molecular tests identify the MTBC and its resistances to key first- and second-line drugs from smear-positive or -negative sputum specimens, although with reduced accuracy compared to DNA isolates from cultures [12]. The real-time PCR-based GeneXpert is capable of identifying MTBC DNA as well as rifampin resistance, mediated by mutations in the rifampin resistance-determining region of the *rpoB* gene, as a “close-to-point-of-care-test” with an assay turnaround time of less than two hours. Yet, all these classical molecular assays are limited because they interrogate only few and confined parts of the genome. Also, they can only be used as rule-in test for drug resistance and do not infer comprehensive drug susceptibility.

## From clinical sample to genome

The advent of WGS in routine microbiological diagnosis allows characterizing all known genes associated with resistance, providing access to the full “resistome” of bacteria of the MTBC. In turn, the absence of any known molecular resistance marker for a certain antibiotic offers the opportunity to predict drug susceptibility, especially for the well-defined first-line drug targets [13]. Ideally, as with the GeneXpert, the infecting MTBC strain is sequenced directly from the patient’s sputum, which, however, remains technically challenging [14]. Using targeted DNA enrichment, resistance prediction based on sequenced genomes from sputum was available within 5 days compared to 36 days from pDST [15]. In this study, the authors obtained whole genomes (defined as >85% single read coverage against the reference genome) in 74% of the sputum samples. When capturing only resistance-associated regions, direct sputum sequencing was obtained within three days of sample receipt [15]. Although this technique requires refining and simplification for use under service conditions, direct sputum sequencing can significantly reduce turnaround time to provide comprehensive genotypic DST results. Another report assessed the turnaround time of sequencing for resistance prediction versus pDST from early-positive Mycobacterial Growth Indicator Tubes that are widely used as liquid culture for MTBC detection. Results from WGS for first-line drugs were available within 72 hours of delivery compared to 28 days on average from pDST [16].

Routinely, the genome sequence of the infecting MTBC strain is obtained from high-quality DNA isolated from cultured organisms. The amount of DNA needed for the sequencing library preparation is specific for the sequencing platform and chemistry used. Upon alignment of the genomic sequence data to a reference TB strain, using bioinformatics software and algorithms, the recorded variants are reviewed and interpreted. These variants comprise mutations of a single nucleotide, as well as deletions or insertions of multiple nucleotides of the infecting strain compared to the reference. Management of a routine WGS workflow and the generated data still requires a dedicated bioinformatics team and server architecture. However, several commercial (such as Applied Maths Bionumerics, Ridom SeqSphere+) as well as freely available software packages (such as CASTB, KvarQ, Mykrobe Predictor TB, PhyResSE, TBProfiler) exist to analyze WGS data with a predefined analysis pipeline and to derive resistance-conferring mutations and lineage classification on the basis of available mutation catalogues [17].

## From genome to resistance prediction and treatment design

For many of the detected mutations, inconclusive genotype-phenotype correlations render the analysis challenging. While this association is straightforward for some drugs, it is more intricate for others, e.g., rifabutin, ethambutol, and pyrazinamide [18,19]. On the one hand, clinical breakpoints to certain antibiotics are significantly higher than the epidemiological cutoff (ECOFF), i.e., highest minimum inhibitory concentration (MIC) of a genotypic wild-type strain. On the other hand, some breakpoints bisect the MIC distribution of mutant strains carrying (low-level) resistance-mediating mutations. Both observations likely lead to the report of false susceptible pDST results and suggest the use of the genotype for resistance to individual drugs as proxy for phenotypic resistance [19]. Vice versa, currently available mutation catalogues likely do not cover the full “resistome” of the MTBC, and high sensitivities to predict resistance or susceptibility to the major first-line drugs would be desirable for a frontline diagnostic test. To fill this gap, global consortia such as CRyPTIC (www.crypticproject.org) and ReSeqTB (platform.reseqtb.org) have been established to increase the performance of molecular predictions of drug resistance in TB. Results from the CRyPTIC consortium of >10,000 genomes found high sensitivities to detect resistant phenotypes for all first-line drugs, i.e., 97.1% for isoniazid, 97.5% for rifampin, 94.6% for ethambutol, and 91.3% for pyrazinamide [20]. Sensitivity to predict susceptibility to the first-line drugs isoniazid and rifampin was 99% and 98.8%, respectively [20]. Recently, a genome-wide association study based on 6,465 clinical TB isolates pointed out several new mutations associated with phenotypic resistance to different MDR-TB and/or XDR-TB drugs [21]. In another recent effort using likelihood ratio thresholds to nominate variants that cause phenotypic resistance with high probability, the authors were able to sort 286 variants from 20 resistance-conferring genes into a grading system comprising three levels—high, moderate, and minimal confidence [13]. In addition, Yadon and colleagues further provided over 300 resistance mediating mutations in the gene *pncA*, which confer pyrazinamide resistance along with a classification of susceptible variants [22]. Independent clinical studies comparing genomic versus traditional pDST data have recently confirmed that treatment regimens designed with WGS data are congruent with those guided by pDST [9,23]. Treatments based on WGS were correctly designed in 93% compared to pDST in a cohort of 25 MDR-TB and/or XDR-TB patients, and only WGS-based regimens contained no drug that was tested resistant by pDST [9].

Genome-based resistance or susceptibility predictions will likely enter the routine diagnostic workflow in the near future. Larger studies will settle nonconclusive genotype-phenotype relations to finally create a work list of highly predictive mutations for clinical use.

## From genome to outcome prediction

It is generally accepted that genetic tests should have pDST as gold standard, although certain pDSTs are known to lack reproducibility due to technical limitations and likely bias the interpretation of some mutations [19]. As a consequence, the real gold standard should be treatment outcome. First clinical data are accumulating, demonstrating how pathogen-based genetic information provides insights on potential treatment course and outcome. Two retrospective studies illustrated that specific codon mutations in the fluoroquinone resistance-conferring *gyrA* gene were linked to poor treatment outcome in MDR-TB patients [1,2]. The presence of these *gyrA* mutations was strongly associated with death or treatment failure after controlling for host and treatment factors in the cohort. A prospective study with 252 patients with culture-confirmed MDR-TB confirmed the role of fluoroquinolone resistance in precipitating treatment failure [3]. Mutations in other resistance-conferring genes have also been shown to impact treatment outcome. Resistance to isoniazid, caused by the specific *katG* codon 315 variant but not *inhA*, was shown to accrete unfavorable outcome [4].

In 2017, England was the first country to implement routine WGS for diagnosis and resistance prediction of MTBC on a national scale [24]. Pioneering WGS at a population level facilitates a scenario in which pDST is no longer required for genetically susceptible strains. These patients can be treated using a first-line regimen, given the well-characterized genotype-phenotype link for these drugs. Ultimately, the data generated by this large-scale sequencing effort will allow us to correlate the presence of resistance-conferring mutations with clinical outcome.

## Outlook and other aspects of personalized therapy for MDR-TB

While genome-based resistance prediction has already been practiced in some TB centers leading to individualized therapy for patients, additional aspects of personalized therapy will likely guide treatment in the future. Firstly, therapeutic drug monitoring using dried blood-spots provides information on the drug level at a certain time point. The physician can modify the dose accordingly to reduce the chance of emerging resistance and mitigate drug-associated side effects [25]. Secondly, the development of reliable biomarkers for diagnosis and treatment monitoring will further personalize treatment. The recent identification of whole-blood—based host genetic signature comprising four genes that predicts progression to TB is promising [26]. Finally, insights on the phylogenetic lineage of the individual and infecting MTBC strain, coupled with their virulence and transmission properties, may inform and further individualize the treatment course. Although highly clonal, there is significant genetic diversity among clinical MTBC strains that translates into relevant biological diversity and versatility of the tubercle bacilli in their respective in vivo niches. Strains of different MTBC lineages can be highly distinct in their host tropism and ability to progress from latency to active disease biology [27]. Drawing on the geographical distribution and restriction of the different lineages, the notion emerged that some strains adapted to their local hosts, leading to a treatment paradigm in which the phylogenetic group of the infecting pathogen needs to be taken into account as a risk factor [28].

## Conclusion

In the absence of horizontal gene transfer, its slow mutation rate, and its highly clonal population structure, *M*. *tuberculosis* infection is the ideal arena to pioneer pathogen-genome guided treatment decisions. WGS has the potential to accelerate the time-consuming and cumbersome diagnosis and resistance profiling of MTBC strains to deliver individualized and effective treatment regimens in a timely manner. Thorough clinical evaluation of treatment is warranted based on as yet unexplained genotype-phenotype correlations. With population-level WGS introduced, England antecedes a diagnostic algorithm in which pDST will only be required in cases of first-line drug resistance in a standardized regimen. Exploiting the entire potential of WGS, detailed information about the phylogenetic lineage of infecting strains can inform the clinician on the readiness of the strain to develop further drug resistance. Together, these innovative approaches in TB treatment herald a new era in treatment of MDR-TB that will contribute to reducing treatment failure and ongoing transmission.
